# Progress in Medicine

**Published:** 1899-08-19

**Authors:** 


### Progress in Medicine.

### DISEASES OF THE BLOOD.

(*Continued from page* 330.)

**Examination of the Blood.**—Louis Fenner[12] recommends a simple solution which fixes and double-stains blood films in two minutes and keeps well. It is derived from the precipitate formed in a watery solution of equal parts of eosin and methylene blue. The author takes Grübler's water soluble eosin (yellow shade) and methylene blue, dissolves them separately in distilled water and mixes the solutions in an open dish. The precipitate is washed and dried twice, and finally dissolved (·5 grm. in 100 c.c.) in methyl alcohol. Cover glass preparations are simply dried and have a few drops of this solution poured on them. They are placed under a watch glass to prevent evaporation for one to three minutes, then rinsed in distilled water for about eight seconds, lightly dried, and mounted in xylol balsam. Red corpuscles appear of a terra-cotta colour, nuclei, blue; granules of basophil cells, violet; those of the eosinophils red; and bacteria or plasmodes, blue. The colouring matter is thus split up, and the acid tissues combine with the basic part and *vice versâ*. It is essential that the cover-slips should be free from any acid or alkali; and to this end, after washing in distilled water, they must be kept in absolute alcohol before using, and distilled water only should be employed in rinsing the specimens. The dried staining material can be obtained ready for use from Kanthack, 18, Berners Street. P. N. Gerard[13] uses the Aronson and Phillips' stain for malaria undiluted, and fixes the specimens by drying them at 120 deg. C. for two minutes. They are then placed in the strong stain for two minutes only, and thoroughly washed till part of the film shows an orange tint. The corpuscles are a very bright orange, and parasites, though almost colourless, show well by contrast. In counting white and red corpuscles A. G. Phear[14] uses a single liquid film, and in order to increase the area needed for a sufficient number of white cells he employs the camera lucida, marking out the squares on a piece of cardboard by the side of the microscope. The image of the cells lying over the unruled part of the stage is thrown on to these squares in succession, and their number counted. As a diluting fluid he finds Sherrington's formula gives good results, preserving the colour and shape of the red cells, and staining the nuclei of the white ones. It is made as follows: Distilled water, 300 grammes; sodium chloride, 1·2; neutral potass. oxalate, 1·2; methylene blue, 1·0; mix.

**Addison's Disease.**—A. G. Auld[15] upholds his original theory that the medulla of the suprarenals is the only active part, and quotes Abel's results in favour of the active principle being an alkaloid with the formula $Cl_{17}H_{15}NO_4$. From recent experiments he argues that this substance, if injected, is destroyed in the blood and organs. Besides, when one suprarenal gland is removed, he finds the spleen becomes enlarged, and the thymus hypertrophied. If both are taken away death follows in one or two days. He concludes that the substance in question is not a secretion, but a poison separated from the blood by the gland, and as a result of its decomposition a vasoconstricting material may be formed in the gland, but not excreted as such. The extraordinary power of the extract of suprarenals in contracting blood-vessels is attested by various writers.[16] It has been used in the eye and on granulating wounds as an astringent, while it has no effect on accommodation. In chloral and chloroform poisoning intravenous injections at once cause recovery in lower animals. It has, however, little effect when given by the mouth, or injected into the subcutaneous tissues, on account of its rapid elimination or destruction. It has been used, however, externally in recent eczema, and, according to several observers, it possesses a marvellous power of stopping the symptoms of hay fever for twelve hours or longer. Dreyer argues that it is a secretion of the gland, and can be found in the adrenal vein; while, as we have seen, Abel[17] has isolated it, determined its percentage composition, and named it Epinephrin. The free base prepared at low pressure, as well as its sulphates and benzoates, is active in producing the effects of the crude gland extract. It is definitely shown to be distinct from pyrocatechin, and now that it can be obtained pure widely extended trials may be looked for, as well as its synthetic preparation. It has been estimated that the glands of 2,000 sheep only contain one drachm of pure Epinephrin, so that a means of synthetic formation is much to be desired.[18] Its formula is similar to sanguinarine, which also possesses the power of raising the blood pressure.

Bates[19] has employed suprarenal extract in dry catarrh of the ear, and for tinnitus due to inflammatory conditions. He quotes also a case of pernicious anæmia where the red cells were markedly increased, and one of Graves' disease under the care of Crary which improved in every respect under its use. Wallace and Mogt[20] found that the extract stimulates the vagus and also the heart muscle directly, as well as producing a marked contraction in the arterioles of the systemic circulation, but not in those of the pulmonary vessels. Destot treated a case of Addison's disease with increasing injections of the extract, and found that the pigmentation rapidly disappeared. The treatment, however, had to be stopped from the condition of the bowels. Carlin Philips[21] discusses a case of the disease where the suprarenals proved to be atrophied, which has been noted in 14 other cases, and, indeed, appears to be the most common cause of the disease apart from tuberculosis. However, J. Levin points out that in 12 per cent. of the cases of Addison's disease the suprarenals are quite normal, so that the connection between the two conditions is uncertain. A well-marked case of the disease in a woman of 38, treated by the extract, is reported by A. Foster.[22] Some relief was noticed, the pulse was quickened, and the pain in the back diminished, and there was less faintness; but the pigmentation remained, and the patient died after a series of convulsions. This temporary improvement has been noticed in some like cases. Others, according to G. Ringer and Phear, are much benefited, while in most instances very little effect is shown.[23] In a patient under the care of Charlewood Turner,[24] where the extract was tried too late to be of service, the blood pressure was measured by the sphygmometer, and the reduction produced by the disease was proved to be great. Instead of being equal to 100 or 125 mm. of mercury, as in health, the tension was only 73 mm. An occasional termination of Addison's disease is insanity. Munson some time ago reported two cases in which depression, dulness, and groundless fears were noticed, and irritability and violence came on later. A. Mclane Hamilton[25] speaks of another where the disease developed in a weak-minded man, and was followed by depression, delusions, and angry outbursts. He refers to two other instances of a like nature, and, in discussing the value of the extract, mentions that he has given it with some success in exophthalmia; while in two cases of hysterical mania it reduced the excitement and caused sleep when 5 to 20 grains were administered in divided doses. Thibierge[26] showed recently an instance of Addison's disease in a negro. The mucous membrane of the mouth was marked by numerous patches of pigment, and the skin had become definitely darker during the last three years. The patient was tuberculous, and complained of lumbar pains and great weakness, as is usually the case.

**Acromegaly.**—Lyon[27] records the treatment of a fat, apathetic patient with Hypophysin. The pulse became regular, the headache, nystagmus, and mental condition were improved, and the weight was reduced by nine kilogrammes. Leszynsky[28] showed a well marked case of the disease in a policeman aged 36, with failing vision, alterations in the visual field, loss of smell and sexual power, and enlargement of phalanges, feet, clavicles, and bones of the head. Oliver Osborne remarked that these cases could be distinguished from pulmonary osteo-arthropathy by the hands alone, for in the latter the fingers are sausage shaped and the nails small. He denied that a large thymus exists in acromegaly, the mass so often found being really a thoracic enlargement of the thyroid gland. Other observers have noticed that the thyroid is enlarged in a number of instances, while the thymus, or what was taken for it, is recorded as persisting in eight patients. The heart, the liver, and spleen are frequently found of abnormal size. An elaborate review of the literature of the subject is given by Mitchell and LeCount,[29] who conclude that true tumours of the hypophysis are not so numerous as has been thought, and the disease does not entirely depend on the loss of function of that organ. The changes in the hypophysis may, they think, be sometimes due to enlargement of the sella turcica, and the cases examined show that some connexion exists between the thyroid and hypophysis. The enlargement of the heart, thyroid and sella turcica is more constant than pathological changes in the hypophysis.

[12] Lancet, Feb. 11. [13] Lancet, June 3. [14] Lancet, May 20. [15] Brit. Med. Jour., Jan. 3. [16] Merck's Archives, Feb. [17] Brit. Med. Jour., Mar. 3. [18] Columbus Med. Jour., Mar. 3. [19] Les Nouveaux Remèdes, April 8. [20] Brit. Med. Jour., Mar. 3. [21] Med. Rec., Mar. 18. [22] Lancet, June 10. [23] Transact. Clin. Soc., Vol. xxix., p. 68. [24] Lancet, June 10. [25] Med. Rec., April 29. [26] New York Med. Jour., Mar. 25. [27] Brit. Med. Jour., Dec. 10. [28] Med. Rec., Mar. 4. [29] New York Med. Jour., April 15, 22, 29.

